# Improving authenticity and provenance in digital biomarkers: the case for digital watermarking

**DOI:** 10.1038/s41746-024-01374-4

**Published:** 2025-01-15

**Authors:** Arjun Mahajan, Dylan Powell

**Affiliations:** 1 Harvard Medical School, Boston, MA USA; 2 Faculty of Health Sciences & Sport, University of Stirling, Stirling, UK

**Keywords:** Biomarkers, Medical research

## Abstract

Enabled by the rapid rise in data collected by technologies, Digital Biomarkers (DBx) have emerged as a novel mechanism for assessment, diagnosis, and monitoring. However, the exponential growth and ability to generate new data has also raised questions about ways of ensuring the authenticity and accuracy of digital data. A recent study highlights how Large Language Models (LLMs) generating human-like content amplify these risks, and propose watermarking as a scalable solution to ensure data integrity. This article examines the potential of digital watermarking to help safeguard the reliability and provenance of DBx data, whilst also addressing broader challenges in health systems.

Over the last two decades, digital innovation and technology has rapidly reshaped health systems, bringing both progress and challenges. While the benefits of these advancements continue to be debated, there is broad agreement on the challenges posed by the exponential increase in the volume, velocity, and variety of data generated by digital health technologies. This surge in data has also given focus to digital measurements and Digital Biomarkers (DBx) quantifiable outcomes derived from data such as images, text, audio, and video, collected via wearable and digital technologies. Digital biomarkers have emerged as a promising paradigm in healthcare, aiding the diagnosis, monitoring, and treatment of various health conditions^1,2^. However, alongside these opportunities come challenges, particularly in ensuring the authenticity and accuracy of the data that underpins DBx. A recent *Nature* study^3^ highlighted these challenges in the context of Large Language Models (LLMs), which are now capable of generating high “quality text often indistinguishable from human written content”. This study emphasized watermarking as a scalable solution to identify synthetic content and prevent accidental or deliberate misuse. Specifically, novel watermarking algorithms were introduced to enable the identification of LLM generated outputs.

These findings have broader implications for areas such as digital DBx, where synthetic or manipulated data could compromise clinical outcomes, research findings, and progress in health innovation. Given the increased focus and reliance of DBx in clinical practice and research, the question arises: should there be a renewed focus on watermarking as a tool to ensure the integrity and provenance of this critical data? In this article, we explore the opportunity for digital watermarking as a potential solution for improving health data integrity, authenticity, and provenance within DBx.

## What is digital watermarking and how does it work?

Digital watermarking applies steganographic principles to embed identification data into digital signals by making imperceptible modifications to redundant or insignificant components. Watermarks can also be designed to be either robust (surviving common modifications) or fragile (breaking upon tampering), with robust watermarks better suited for ownership proof while fragile ones excel at tamper detection. This verification mechanism also supports continuous authentication, rather than discrete point-in-time checks like two-factor authentication. Current applications of watermarking also include securing authenticity in Portable Document Format files and safeguarding intellectual property in video and multimedia streaming by preventing unauthorized content distribution^4,5^.

**Fig. 1:**
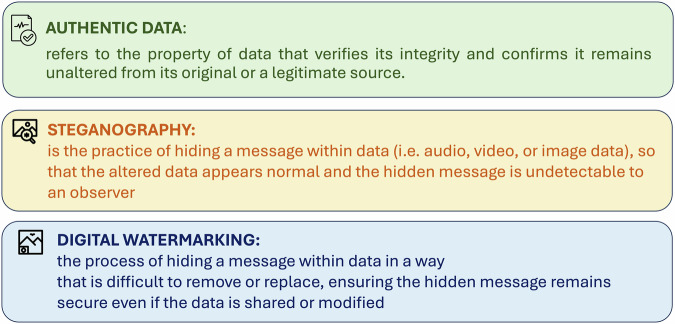
Key elements in data integrity and security. This figure outlines the description of authentic data, steganography and digital watermarking.

**Fig. 2:**
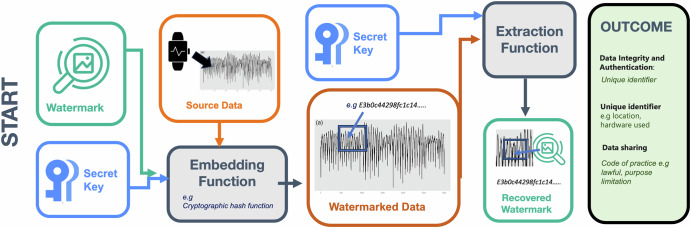
Watermarking in digital biomarkers. Example overview of digital watermarking process within gait data.

This technique is particularly useful for inherently noisy signals like audio or image data, where small modifications can be hidden within the natural variations of the signal (Figs. 1, 2).

In digital biomarkers, this could mean in gait data collected via wearable sensors, watermarks may be integrated without altering the core functionality, ensuring authenticity and traceability.

New technological approaches, such as blockchain-based protocols, are also emerging in implementing watermarking-based copyright and purchase transaction protection mechanisms^6,7^. Similarly, by enabling robust verification processes, watermarking may help identify and signal any signs of alteration, ensuring that health data remains reliable.

## What might be some of the benefits?

### Ensuring proof of ownership and authenticity

Digital watermarking offers a practical opportunity and solution to better verify and protect health data throughout its lifecycle addressing research, clinical, and regulatory aspects. For example, in clinical practice or in its primary use, watermarked vital sign data or acoustic (voice) measurements may provide clinicians, or other stakeholders greater evidence that the readings were not created synthetically and are “authentic”.

In secondary uses, such as research and innovation, watermarking may play a unique role in ensuring the traceability and integrity of datasets. Unlike encryption or secure checksum approaches, watermarking embeds provenance information directly into the data, allowing for ongoing verification even after data has been transferred, or shared across platforms. This capability is particularly valuable in algorithm audits, as highlighted by regulatory frameworks like the UK’s DRCF Algorithmic Processing Workstream, which emphasizes the need for transparency and accountability in data usage^8,9^.

### Data provenance and traceability

Beyond preserving data integrity, watermarking may also provide a way to enhance transparency and empower patients by giving them greater control over their health data. Research into tools like the NHS mobile application highlights efforts to let patients manage how and why their data is shared^10^. Watermarking could complement such systems by ensuring data provenance remains intact during transfers or copies. Unlike metadata tracking or audit logs, watermarking integrates directly with the data, making it more resistant to tampering or loss. This ensures robust verification of data use and origins, fostering trust between patients and providers. By offering granular visibility into data usage, watermarking can help identify unauthorized access or modifications, reinforcing confidence in digital health systems. It also supports meaningful discussions between patients and providers about data use, enabling patients to play a more active, informed role in their care as digital tools increasingly shape healthcare delivery.

## Considerations moving forward

Despite its promise, digital watermarking in healthcare comes with both technical and practical challenges.

### Technical and implementation-related considerations

There is a need to focus on ensuring watermark durability, as current practices for multimodal data processing involve compression (e.g., lossless versus lossy compression), encryption, or transfers across platforms, which may promote degradation or loss of the embedded watermark^9,10^. Digital watermarking and watermark resilience against common data transformations may also require standardization for compatibility across diverse Electronic Health Record or research systems. Furthermore, scalability and efficiency challenges arise as watermarking algorithms must should be optimized to handle vast amounts of real-time data across large healthcare networks without slowing data flow or compromising functionality. Adaptive watermarking technologies that extend beyond the point of data generation are particularly important for DBx, such as gait data collected via wearable sensors. For instance, gait analysis used to monitor conditions like Parkinson’s disease or post-stroke rehabilitation may benefit from embed traceability directly into the time-series data^11^.

### Patient autonomy and ethical standards

Perhaps most crucially, the watermarking process must be carefully designed to balance the benefits from data authenticity verification with the need for stringent privacy protections, safeguarding sensitive patient information, and respecting patient preferences, including scenarios where individuals may not want their data tracked or monitored^12,13^. To strengthen patient autonomy, there may be opportunity in creating accessible solutions for patients. This may require developing intuitive platforms that allow individuals to track who has accessed their health data, review permissions, and make informed decisions about sharing or restricting data access. Additionally, revisiting and establishing ethical guidelines or best practices around the use of watermarking in medical research and public health surveillance, defining appropriate de-identification standards and preventing the system from inadvertently creating new forms of healthcare discrimination by making certain populations’ data more traceable than others^12,13^.

### Harmonization with existing principles, standards, and ecosystem

Digital watermarking must not only align with foundational security, privacy and research standards like the Health Insurance Portability and Accountability Act, Digital Object Identifier, and FAIR Principles but should also actively support health systems in maintaining compliance with these frameworks by creating an *embedded* auditable trail of patient data access and modifications^14^.

Watermarking can ensure traceability and authenticity but this depends on the context of use. In closed systems, it may primarily safeguard against tampering and unauthorized distribution rather than providing transparency to patients or stakeholders. For publicly shared datasets, watermarking verifies provenance and detects manipulation, proving particularly useful in secondary applications like AI model training or research. To achieve greater transparency in situations where data usage occurs behind closed doors, watermarking should be paired with complementary tools like audit logs, regulatory oversight, and lineage reporting mechanisms. Together, these measures provide a comprehensive framework for safeguarding data integrity, ensuring compliance with standards, and fostering trust by making data lineage more transparent to both patients and stakeholders.

## Conclusion

As digital watermarking evolves, it may become a key enabler of reliable and patient-centered and decentralised health. By tracking data provenance and ensuring data integrity, watermarking technology may help build trust in an increasingly algorithm driven healthcare ecosystem. However, challenges remain such as ensuring watermark durability, standardization across clinical and research systems, and addressing ethical concerns. Continued research and thoughtful implementation may overcome these hurdles and unlock the full potential of watermarking for improved digital health data authenticity and provenance.

## Data Availability

No datasets were generated or analyzed during the current study.
